# Mechanism of hif-1α mediated hypoxia-induced permeability changes in bladder endothelial cells

**DOI:** 10.1590/1414-431X20176768

**Published:** 2017-12-18

**Authors:** C. Liu, C.L. Shui, Q. Wang, H. Luo, C.G. Gu

**Affiliations:** 1 Department of Urology, The Second People's Hospital of Deyang City, Deyang, Sichuan Province, China; 2 Department of Anesthesiology, Affiliated Yongchuan Hospital of Chongqing Medical University, Yongchuan, Chongqing, China; 3 Department of Pathology, Cancer Center of Guangzhou Medical University, Guangzhou, Guangdong Province, China; 4 Department of Laboratory Medicine, The Fifth People's Hospital of Chengdu, Chengdu, Sichuan Province, China

**Keywords:** Bladder cancer, Hypoxia inducible factor-1α, VEGF, Cell permeability, YC-1

## Abstract

This study aimed to investigate the mechanism of hypoxia-inducible factor-1 alpha (HIF-1α) mediated hypoxia-induced permeability changes in bladder endothelial cells. Models of *in vitro* hypoxic cell culture of bladder cancer, bladder cancer cells with low HIF-1α expression and HIF-1α RNA interference (RNAi) expression vector were established. Western blot and reverse transcription polymerase chain reaction (RT-PCR) were used to detect the expression of HIF-1α and vascular endothelial growth factor (VEGF) in each group. Bladder cell permeability was determined. Results showed that protein and mRNA expression of HIF-1α and VEGF at 3 and 12 h of hypoxia were significantly higher than normal control (P<0.05), and peaked at 12 h. HIF-1α and VEGF expression in the hypoxic group and hypoxic+3-(5′-hydroxymethyl-2′-furyl)-1-benzyl indazole (YC-1) group were significantly higher than normal control (P<0.05), while expression in the hypoxic+YC-1 group was significantly lower than the hypoxic group (P<0.05). Bladder cell permeability in the hypoxic and hypoxic+YC-1 group were significantly increased compared to normal control (P<0.05), while in the hypoxic+YC-1 group was significantly decreased compared to the hypoxic group (P<0.05). Most of the cells in the stably transfected HIF-1α RNAi expression vector pcDNA6.2-GW/EmGFP-miR-siHIF-1α expressed green fluorescence protein (GFP) under fluorescence microscope. pcDNA6.2-GW/EmGFP-miR-siHIF-1α could significantly inhibit HIF-1α gene expression (P<0.05). HIF-1α and VEGF expression in the hypoxic group and siHIF-1α hypoxic group were significantly higher than normal group (P<0.05), while expression in the siHIF-1α hypoxic group was significantly lower than the hypoxic group (P<0.05). Findings suggest that HIF-1α is an important factor in the increase of bladder cancer cell permeability.

## Introduction

Bladder cancer is the most common malignancy of the urinary tract, having a high incidence rate and a recurrence rate above 80%. Patients not only have to bear the burden of high medical expenses, but also the stress of psychological distress caused by relapses. Non-muscle invasive bladder cancer (NMIBC) comprises about 70% of all newly diagnosed bladder cancers. NMIBC tumor stages include papillary lesions that have not invaded sub-mucosal lamina propria (Ta), papillary lesions that have invaded the lamina propria but not the muscle (T1), and carcinoma in situ (CIS). At present, transurethral resection followed by instillation of adjuvant intravesical chemotherapy has been the main treatment of choice for NMIBC. However, NMIBC still has a recurrence rate of 70–80% (1,2).

Tumor cells regulate and adapt to the hypoxic environment by initiating a series of hypoxic adaptive responses. A number of studies reported that hypoxia-inducible factor-1 (HIF-1) is the central mediator of adaptive responses to hypoxia in tumors. HIF-1 is a heterodimer transcription factor consisting of α and β subunits. Among these, HIF-1α determines the activity of HIF-1 and is the sole oxygen regulator. HIF-1 can adapt to hypoxia by regulating the expression of various target genes and involves in the process of tumor growth, invasion and metastasis (3). HIF-1α has been found to be highly expressed in a variety of malignant tumors and precancerous lesions, indicating that it is closely related with tumor angiogenesis, invasion and metastasis (4).

The synthetic compound hypoxic+3-(5′-hydroxymethyl-2′-furyl)-1-benzyl indazole (YC-1) has superior anti-angiogenic and anti-tumor activities. YC-1 was first discovered by Ko et al. (5), who reported that its role is achieved by activating platelet soluble guanylate cyclase and upregulation of adenosine monophosphate and cyclic guanosine monophosphate. Vascular endothelial growth factor (VEGF), also known as vascular permeability factor, is an efficient and specific growth factor for vascular endothelial cells. It has a strong effect on promoting mitosis and chemotaxis of vascular endothelial cells, stimulating angiogenesis, and increasing vascular permeability. In normal tissue, its expression is very low or absent, but it is highly expressed in malignant solid tumors and positively correlated with the malignancy grade of the tumor (6). This paper describes for the first time the role of HIF-1α/VEGF in cancer.

This study aimed to investigate the role of HIF in the recurrence of bladder cancer at the gene level, in order to provide a new therapeutic approach for the disease. We hypothesized that during hypoxia, an increase in the HIF-1α and VEGF protein expression would lead to an increase in bladder cancer cell permeability, and that HIF-1α is an important factor in the increase of bladder cancer cell permeability.

## Material and Methods

Experiments were performed at the Central Laboratory of the Affiliated Yongchuan Hospital of Chongqing Medical University and Department of Pathology, Cancer Center of Guangzhou Medical University. Cells collection period were from February 2015 to February 2016.

All procedures were approved by the Ethics Committee of The Second People's Hospital of Deyang City, Sichuan, China (Reference number [2014]-20) and in accordance with the 1964 Helsinki declaration and its later amendments or comparable ethical standards.

### Material

#### Cell lines

Bladder cancer cells were purchased from Cell Bank of the Chinese Academy of Sciences (Shanghai, China).

#### Main reagents and instruments

Rabbit HIF-1α polyclonal antibodies (Upstate Biotechnology, USA); VEGF polyclonal antibodies, albumin-fluorescein isothiocyanate conjugate (FITC-albumin), β-actin monoclonal antibodies (Sigma-Aldrich Corporation, USA); IgG secondary antibodies (Beijing Zhongshan Gold Bridge Biotechnology Corporation Ltd, China); newborn calf serum (Chengdu Harry Biotechnology Corporation Ltd, China); primer synthesis (Sangon Biotechnology (Shanghai) Corporation Limited, China); Roswell Park Memorial Institute (RPMI) 1640 culture medium (HyClone Laboratories, USA); Bicinchoninic acid (BCA) protein assay kit (Pierce^TM^, Thermo Fisher Scientific, USA); Mini Trans-Blot® electro transfer system, fluorescence quantitative PCR instrument, ChemiDoc XRS Gel Analysis System, “Quantity one” software (Bio-Rad Laboratories, USA); Milicell^TM^ electrical resistance system (ERS) instrument (Millipore, USA); inverted fluorescence microscope model TH4-200 (Olympus Corporation, Japan); ultra-sensitive enhanced chemiluminescence (ECL) kit (Pierce Biotechnology, USA); HeraeusCO_2_ constant temperature incubator (Heraeus, Germany); DU®-640 ultraviolet spectrophotometer (Beckman Inc., USA); modular incubator chamber (Billups-Rothenberg Inc., USA).

### Methods

#### Model of *in vitro* hypoxic cell culture of bladder cancer

Bladder cancer cells were cultured in a culture flask with primary culture medium and kept at 37°C in a 5% CO_2_ incubator. The culture medium was replaced every 2 days. When the cell culture reached around 90% confluence, it was digested with 0.25% trypsin-0.01% ethylenediaminetetraacetic acid (EDTA) solution and subcultured. Cells were then seeded at a concentration of 1.5×105/cm2 in a 0.1% gelatin-coated Transwell^TM^ polycarbonate membrane. Culture medium was replaced every 2 days after 24 h of inoculation. The transendothelial electrical resistance (TER) of the monolayer endothelial cells was measured with Milicell^TM^ ERS for cell integrity. When the TERs were stable, we proceeded to the next step of experiment. Cells seeded in the 6-well plates were examined and placed in a hypoxic tank filled with 1% O_2_ of mixed gas (94% N_2_, 5% CO_2_, 1% O_2_) and cultured in a 37°C cell culture incubator. Observation was done at 0, 3, and 12 h post-hypoxia.

#### Model of hypoxic HIF-1*α* inhibitor

After the bladder cancer cells in the 6-well plate reached complete confluence or the monolayer bladder cancer cells in the Transwell^TM^ had been tested for TER stabilization, these were randomly assigned into normal control group (normal culture under normoxia with 5% CO_2_ and O_2_ in air), hypoxic group (cultured in 1% O_2_ for 6 h) and hypoxic+3-(5′-hydroxymethyl-2′-furyl)-1-benzyl indazole (YC-1) group (addition of 100 μM YC-1, followed by incubation in 1% O_2_ for 6 h). Time of incubation with YC-1 was 48 h.

#### Model of HIF-1*α* RNA interference (RNAi) expression vector

RNAi expression vector was established. Amplification of recombinant plasmid and DNA extraction, plasmid transfection, screening of SiHIF-1α positive cell clones and identification of pcDNA6.2-GW/EmGFP-miR-siHIF-1α stable expression cell lines were performed.

#### HIF-1*α* and VEGF protein expression (by western blot)

Cells from each group were treated with cell lysate, lysed for 30 min and centrifuged at 8,000–10,000 *g* for 10 min at 4°C. The supernatant was carefully extracted for total protein concentration, which was determined according to the BCA kit instructions. This was followed by protein denaturation, sample uploading, sodium dodecyl benzene sulfonate (SDBS) gel electrophoresis for 1–2 h and wet transfer for 30–50 min. This was then incubated with rabbit HIF-1α and VEGF polyclonal antibodies at 4°C overnight and IgG secondary antibodies at room temperature for 1–2 h. Following this, ECL exposure solution was added and exposure was done under the gel imaging system. “Quantity one” software was used to analyze the gray scale value of each antibody strip.

#### HIF-1*α* and VEGF mRNA expression (detected with RT-PCR)

RNA was extracted with TRIzon, followed by reverse transcription and detection with RT-PCR. Expression ratio and relative content of the HIF-1α, VEGF mRNA and internal reference (β-actin) was calculated. Primers used were as follows: HIF-1α-F: TCAAAGTCGGACAGCCTCA, HIF-1α-R: CCCTGCAGTAGGTTTCTGCT; VEGF-F: GAAGTGGTGAAGTTCATGGATGTC, VEGF-R: CGATCGTTCTGTATCAGTCTTTCC; β-actin-F: TGAGACCTTCAACACCCCAG, β-actin-R: GCCATCTCTTGCTCGAAGTC.

#### Determination of bladder cancer cell permeability

Culture medium was discarded. FITC-albumin (100 μL, 1 mg/mL dissolved in D-Hank solution) was added to the upper part of the double-chamber, while 500 μL D-Hank solution was added to the lower part at 37°C. Then, the culture plate was placed back into the incubator and continued to culture for 1 h. Following this, the solutions from the upper and lower chambers were sucked out.

The excitation and emission wavelength of the fluorescence spectrophotometer were set at 490 and 525 nm, respectively. Fluorescence intensity of each sample was determined to analyze permeability changes.

### Statistical analysis

Data are reported as means±SD. Analysis was performed using SPSS 19.0 statistical software (SPSS Inc., USA). Comparison between groups were performed with ANOVA test. P<0.05 was considered statistically significant.

## Results

### Effects of hypoxia on HIF-1**α** and VEGF expression in bladder endothelial cells

**Figure 1.**
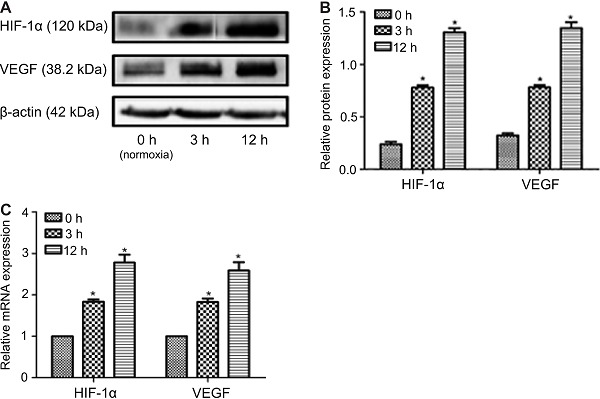
*A*, HIF-1α and VEGF protein expression. *B*, Histogram of HIF-1α and VEGF protein expression. *C*, Histogram of HIF-1α and VEGF mRNA expression. Data are reported as means±SD. *P<0.05 compared with normal control group (ANOVA).

Compared with the normal control group, the protein and mRNA expression of HIF-1α and VEGF were significantly higher at 3 and 12 h of hypoxia, and peaked at 12 h (P<0.05; Figure 1A-C).

### YC-1 reduced the protein and mRNA expression of HIF-1**α** and VEGF expression

**Figure 2.**
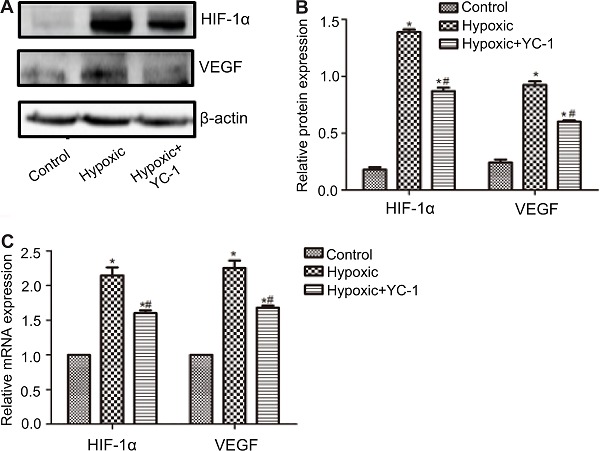
*A*, HIF-1α and VEGF expression. *B*, Histogram of HIF-1α and VEGF protein expression. *C*, Histogram of HIF-1α and VEGF mRNA expression. Data are reported as means±SD. *P<0.05 compared with normal control group; #P<0.05 compared with hypoxic group (ANOVA). Time of incubation with YC-1=48 h.

Protein and mRNA expression of HIF-1α and VEGF in the hypoxic group and hypoxic*+*YC-1 group were significantly higher than the normal control group (P<0.05), while expression in the hypoxic+YC-1 was significantly lower than the hypoxic group (P<0.05; Figure 2A–C). Protein and mRNA expression of HIF-1α and VEGF were significantly reduced by YC-1 (Figure 2B and C).

### Bladder cancer cell permeability

**Figure 3.**
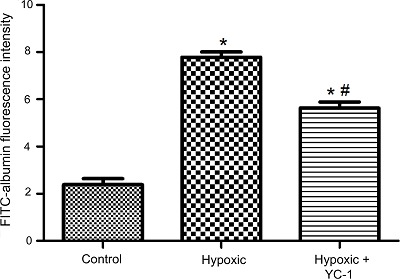
Effect of YC-1 on increased bladder cancer cell permeability by hypoxia. Data are reported as means±SD. *P<0.05 compared with normal control group; #P<0.05 compared with hypoxic group (ANOVA). Time of incubation with YC-1=48 h.

Cell permeability in the hypoxic group and hypoxic+YC-1 group were significantly increased compared to the normal control group (P<0.05), while in the hypoxic+YC-1 group, it was significantly decreased compared to the hypoxic group (P<0.05; Figure 3).

### Effects of HIF-1**α** RNAi expression vector on VEGF expression and bladder cancer cell permeability

**Figure 4.**
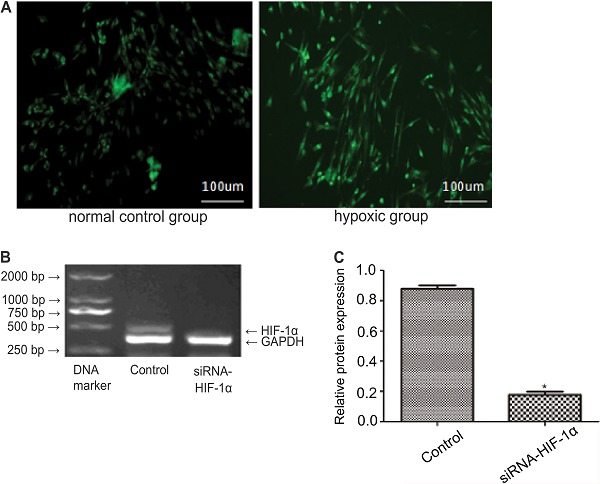
Effects of HIF-1α RNAi expression vector on VEGF expression and bladder cancer cell permeability. *A*, Stably transfected HIF-1α RNAi expression vector in normal control group and hypoxic group. *B*, HIF-1α gene expression after stable transfection of HIF-1α RNAi expression vector pcDNA6.2-GW/EmGFP-miR-siHIF-1α. *C*, Histogram of HIF-1α gene expression after stable transfection of HIF-1α RNAi expression vector pcDNA6.2-GW/EmGFP-miR-siHIF-1α. Data are reported as means±SD. *P<0.05 compared with normal control group (ANOVA). GADPH: glyceraldehyde 3-phosphate dehydrogenase.

Most of the cells in the stably transfected HIF-1α RNAi expression vector pcDNA6.2-GW/EmGFP-miR-siHIF-1α expressed green florescence protein (GFP) (Figure 4A). This vector could significantly inhibit HIF-1α gene expression (P<0.05; Figure 4B and C).

### Effects of stable transfection on HIF-1**α** and VEGF expression

**Figure 5.**
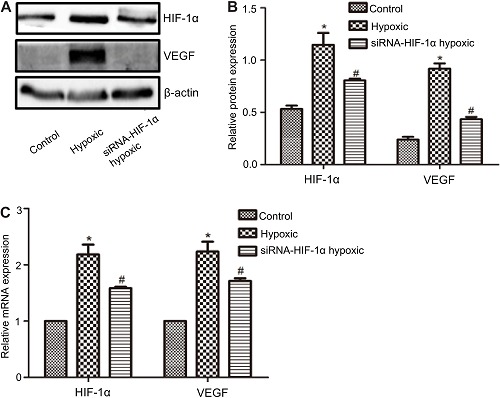
Effects on HIF-1α and VEGF expression after stable transfection. *A*, HIF-1α and VEGF expression. *B*, Histogram of HIF-1α and VEGF protein expression. *C*, Histogram of HIF-1α and VEGF mRNA expression. Data are reported as means±SD. *P<0.05 compared with normal control group; #P<0.05 compared with hypoxic group (ANOVA).

The HIF-1α and VEGF expression in the hypoxic group and siHIF-1α hypoxic group was significantly higher (P<0.05) than the normal control group (Figure 5A). The protein (Figure 5B) and mRNA expression (Figure 5C) in the siHIF-1α hypoxic group were significantly lower than the hypoxic group (P<0.05).

## Discussion

Bladder cancer is one of the most common urologic cancer with high incidence rate. During development, many solid tumors loose regulation of cell proliferation and apoptosis. This accelerates its growth, and when growth rate is higher than the process of angiogenesis, local ischemia and hypoxia of the tumor can occur.

HIF-1a was first discovered by Semenza et al. (7) in 1992. A large number of studies showed that HIF-1α, which has long been recognized as one of the important transcription factor that mediate cellular response to hypoxia, plays an important role in the regulation of pathophysiological changes in ischemic and hypoxic conditions (8). The expression of HIF-1 is subjected to hypoxia, tumor genes and various regulatory factors, and is an important factor in hypoxia-induced adaptive gene expression.

HIF-1 is composed of two subunits, the HIF-1α and HIF-1β subunits. The intracellular protein level of the HIF-1β subunits is not affected by the level of oxygenation while the HIF-1α gene is subjected to the regulation of hypoxia signals. Under normoxic conditions, the α subunit undergoes post-translational modifications and O_2_-dependent prolyl hydroxylation, and is degraded via the ubiquitin-proteasome pathway mediated by pVHL (von Hippel-Lindau tumor suppressor protein). In hypoxic condition, HIF-1α does not undergo self-hydroxylation and degradation, and thus it is stable. It forms a transcriptionally active heterodimer complex with the HIF-1β subunit and binds to the hypoxia-response element of the target genes. It involves the regulation of adaptive response to hypoxia through a variety of target gene expressions (9–11). YC-1 is a specific inhibitor of HIF-1α. It can reduce the accumulation of HIF-1α and inhibit HIF-1 transcriptional activity through a pathway that is independent of soluble guanylate cyclase (12). It can also promote protein degradation of HIF-1α (13).

Vascular endothelium is the inner lining of blood vessels with semi-selective permeable membrane. Endothelial cells regulate the tension of the blood vessel, maintain their normal structure, secrete fibrinolytic protein, anticoagulant and other substances, and have anti-inflammatory effects (14). Recent studies have confirmed that there are many factors involved in the whole process of angiogenesis in solid tumors. Among these, VEGF is known to be the most active and the most specific angiogenesis inducing factor. The increase of HIF-1α and VEGF were parallel, indicating a correlation between the two. Thus, it is speculated that HIF-1α may stimulate angiogenesis by increasing the expression of VEGF to adapt to the growth environment of hypoxic and low-sugar content, so that the tumor can continue to grow and metastasize distantly (15).

A number of studies has identified high expression of HIF-1α in gastric cancer and various other cancers, and it is found to be closely associated with the biological behavior of tumors (16,17). HIF-1α shows high expression in hypoxic environment. This stimulates the release of VEGF in tumor cells and promotes angiogenesis (18,19).

RNAi can silence the expression of target genes. It can specifically knock down or turn off the expression of a specific gene, participate in the protection of genome against invasion and thus maintain the genome stability. This is a technique of reverse genetics, which plays an important role in functional genomics research (20–25).

In the first part of this study, a model of *in vitro* hypoxic cell culture of bladder cancer was established. We preliminarily postulated that increase in HIF-1α and VEGF expression is one of the mechanisms that leads to the increase of cell permeability in hypoxia. In the second part of the study, an HIF-1α inhibitor YC-1 was added and we found that a decrease in the HIF-1α expression resulted in a decrease in VEGF expression and an increase in the permeability of bladder cancer cells during hypoxia. To further clarify whether the activation of HIF-1α was involved in the increase of bladder cancer cells permeability during hypoxia, we used an established bladder cancer cell model with low HIF-1 expression to study the changes of tumor cell permeability in hypoxia.

This study finally found that hypoxia induced an increase in bladder cancer cell permeability. HIF-1α inhibitor YC-1 and HIF-1α RNAi could significantly inhibit the increase of hypoxia-induced cell permeability. HIF-1α is an important factor in the increase of bladder cancer cell permeability.
